# Prevalence of Non-Tuberculous Mycobacterial Infections among Tuberculosis Suspects in Nigeria

**DOI:** 10.1371/journal.pone.0063170

**Published:** 2013-05-09

**Authors:** Gambo Aliyu, Samer S. El-Kamary, Alash’le Abimiku, Clayton Brown, Kathleen Tracy, Laura Hungerford, William Blattner

**Affiliations:** 1 Institute of Human Virology, University of Maryland School of Medicine, Baltimore, Maryland, United States of America; 2 Department of Epidemiology and Public Health, University of Maryland School of Medicine, Baltimore, Maryland, United States of America; Beijing Institute of Microbiology and Epidemiology, China

## Abstract

**Background:**

Nigeria is ranked in the top five countries for tuberculosis deaths worldwide. This study investigated the mycobacterial agents associated with presumptive clinical pulmonary tuberculosis (TB) in Nigeria and evaluated the pattern and frequency of mycobacterial infections over twelve calendar months period.

**Methods:**

Sputum samples from 1,603 consecutive new cases with presumptive diagnosis of TB were collected from August 2010 to July 2011. All sputum samples were incubated for detection of mycobacterial growth and those with positive acid fast bacilli (AFB) growth were tested to detect *mycobacterium tuberculosis* (*MTB*) *complex* and characterized to differentiate between *MTB complex* species. Cultures suggestive of Non-tuberculous mycobacterial infections (*NTM)* were sub-cultured and characterized.

**Results:**

Of the 1,603 patients screened, 444 (28%) culture-positive cases of pulmonary tuberculosis were identified. Of these, 375 (85%) were due to strains of *MTB complex* (354 cases of *M. tuberculosis*, 20 *M. africanum* and one case of *M. bovis*) and 69 (15%) were due to infection with *NTM*. In contrast to the *MTB complex* cases, the *NTM* cases were more likely to have been diagnosed during the calendar months of the Harmattan dust season (OR = 2.34, 1.28–4.29; p = 0.01), and aged older than 35 years (OR = 2.77, 1.52–5.02, p = 0.0007), but less likely to have AFB identified on their sputum smear (OR = 0.06, 0.02–0.14, p<0.0001). Among those with *NTM* infection, cases 35 years or younger were more likely to have co-infection with HIV (3.76, 1.72–8.22; p = 0.0009) compared to those older than 35 years.

**Interpretation:**

The high proportion of younger patients with clinical pulmonary TB due to *NTM* and co-infection with HIV and the likely role of the seasonal dust exposure in the occurrence of the disease, present novel public health challenges for prevention and treatment.

## Introduction

The frequency of pulmonary disease from non-tuberculous mycobacterium (NTM) is reportedly on the rise in Europe, North America, Asia and Southern Africa [1]. In sub-Saharan Africa, information on the extent of the burden of pulmonary disease from non-tuberculous mycobacteria (*NTM*) is lacking due to limitations in tools for mycobacterial species identification. However, studies conducted as far back as the late 1950s and early 1960s using traditional tools for identifying mycobacterial groups based on certain characteristics like speed of growth and morphology, have reported the isolation of *NTM* from both tuberculosis patients and the general public in some African countries including Nigeria. [2], [3].

Failure to characterize acid fast bacilli (AFB) positive *NTM* lung infections has led to their misclassification and to mistake in treatment for pulmonary tuberculosis in developing countries. A recent report from Nigeria found that 12.4% (12/97) of AFB positive patients receiving treatment for pulmonary tuberculosis (TB) had infections with organisms other than Mycobacteria while 4.1% (4/97) had *NTM* infections [4]. In general, *NTM*s are increasingly being isolated among HIV positive and negative cases of TB in Sub-Saharan Africa [5], [6], [7]. A prospective evaluation of a cohort of 721 HIV positive patients in Abidjan, Cote d’Ivoire, Sub-Saharan Africa found the incidence of *NTM* infection was 9.7 times higher among patients with baseline CD4 cell counts less than 100 cells/mm^3^ compared to patients with CD4 cell counts above 100 cells/mm^3^. [8] In addition to HIV disease, *NTM*s have been reported to be more common among persons with occupational exposure to dust [9], [10]. Occupational dust exposure in HIV positive subjects accelerates the risk of pulmonary infection with both TB and *NTM.* [11], [12]. In addition, the risk of reactivation of latent mycobacterial infections including *NTMs* is found to be higher in patients receiving treatment with tumor necrosis factor inhibitors like infliximab and etanercept and this is becoming more prevalent in Europe and United States [13].

Given the high prevalence of HIV infection in Nigeria, there is a growing concern that *NTM* and other infections could be misdiagnosed as pulmonary TB in HIV infected persons. This study reports the prevalence of *NTM* mycobacterial infections among TB *suspect*s and examines factors that differ between *NTM* and *MTB* cases of pulmonary mycobacterial infections in Nigeria.

## Methods

### Ethics Statement

Written informed consents were obtained from all eligible participants. The protocol for this study was reviewed and approved by the University of Maryland’s Institutional Review Board and the Nigerian Health Research Ethics Committee (NHREC).

New patients, 18 years or older previously untested for HIV with symptoms of presumptive pulmonary tuberculosis (based on WHO screening criteria) were consecutively recruited at the National TB and Leprosy Training Center (NTBLTC), Zaria, from August 2010 through July 2011, and at the Barau Dikko Hospital (BDH), in Kaduna City, from December 2010 through July 2011. Participants provided informed consent, responded to an itemized survey instrument and provided three sputum samples: (1) spot sample in the clinic the same day, (2) early morning home-collected sample and (3) spot sample in the clinic the next day. HIV status was determined by a serial rapid assay algorithm using Trinity Biotech Unigold and Abbott Determine.

#### Smear microscopy

Smears of size 1×2 cm were made on new grease-free slides and allowed to air dry. The air dried smears were then fixed by gently passing them over a flame 2–3 times. The smears were then stained with Ziehl- Neelsen (ZN) technique as previously described [14], [15], [16]. Positive and negative controls were included in the staining process. Slides were examined at x100 microscopic magnification. For the purpose of this study, smears were graded as positive when five or more bacilli were detected in at least 100 microscopic fields.

#### Mycobacterial culture and characterization

Of the three sputum samples collected, only the early morning home-collected smear sample was cultured. This sample was selected as the most likely to yield the highest concentration of tubercle bacilli, and the least likely to be contaminated with other bacteria as it was not manipulated to provide the smears used in the routine care ZN staining and microscopic examination. The early morning sputum samples were treated with BD Mycoprep™ (Beckton Dickinson Diagnostic Systems, Sparks, Maryland, USA) which consists of 1% N-acetyl-L-cysteine (NALC), 4% sodium hydroxide and 2.9% then incubated in the automated BACTEC MGIT 960™ machine (Becton Dickinson Diagnostic Instrument Systems) as previously described [17]. Samples that failed to show any growth after 42 days of incubation in the machine were removed and classified as negative based on the manufacturer’s protocol.

Samples with positive growth were removed from the machine and inoculated on blood agar to check for non-mycobacterial contamination. Then, a ZN stain was performed to check for the presence of AFB. Samples without AFB were considered contaminants and excluded from the study. Samples that did not grow were re-incubated for a maximum of 42 days and then classified as above based on the ZN test.

Cultures with positive growth on the BACTEC MGIT and presence of AFB by ZN stain were tested with a rapid TB antigen assay (SD-Bioline Ag MPT64 Rapid™ assay; Standard Diagnostics, Kyonggi-do, Korea) which identifies antigen specific to the *Mycobacterium tuberculosis* complex (*MTB*) group. Samples confirmed as *MTB* isolates were then characterized with the PCR based Genotype *MTB*C test (Hain Lifescience, Nehren, Germany) to identify the strains involved.

**Figure 1:**
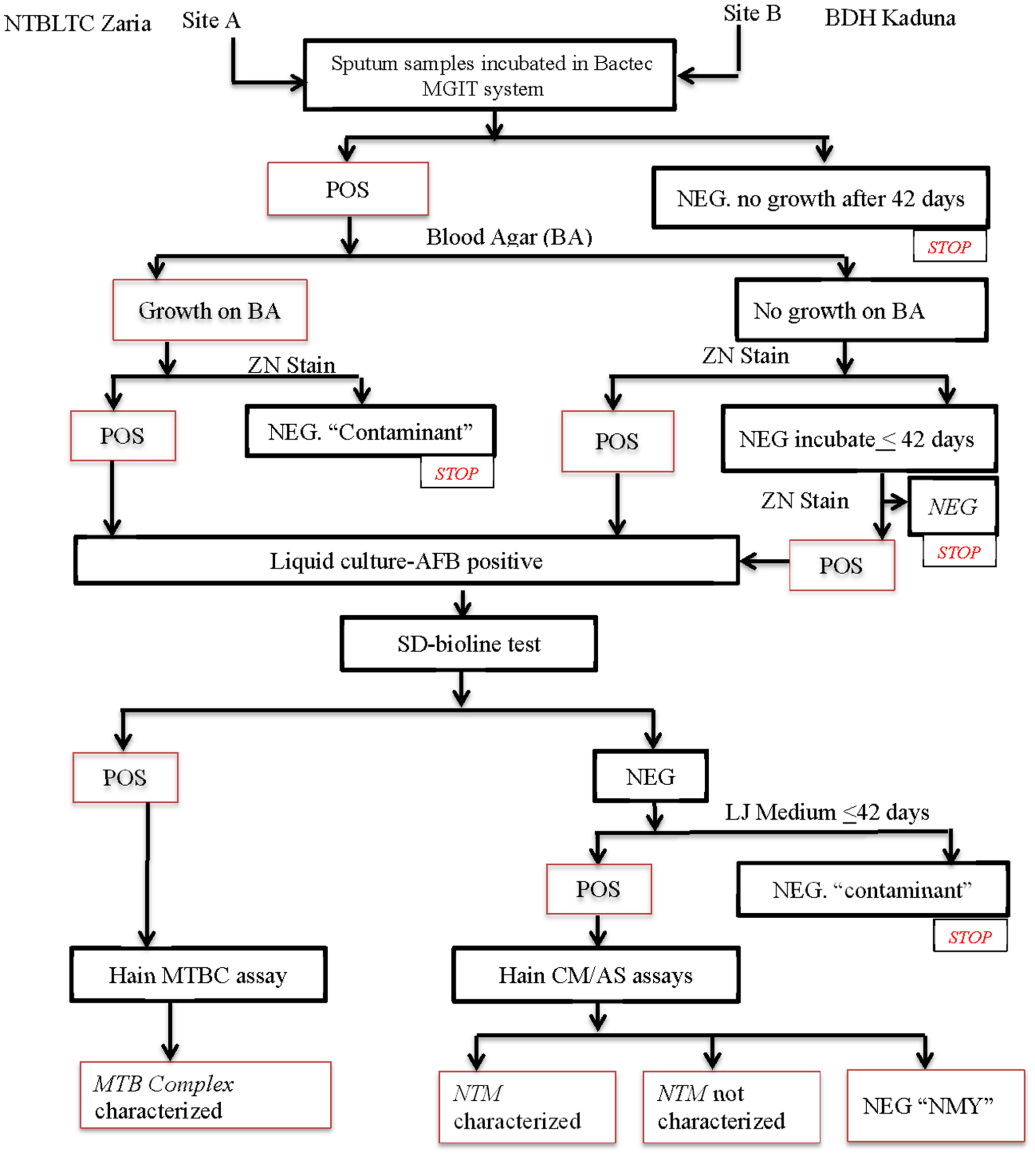
Mycobacterial Detection and Characterization. POS, positive; NEG, negative; ZN, Ziehl Neelsen; AFB, acid fast bacilli; NMY, not of the genus mycobacterium.

Cultures with positive growth on the BACTEC MGIT and presence of AFB but that were negative for *MTB* complex using the SD-Bioline assay were sub-cultured in Lowenstein Jensen (LJ) media. Those that subsequently grew on LJ medium were considered to be *NTM*s and were characterized with the PCR based Genotype CM/AS assays. Thus, the individuals in this study who visited the TB testing centers were divided into four groups: *MTB* cases, *NTM* cases, NMY (not mycobacterium though positive for AFB) cases and those negative for AFB due to lack of bacterial growth or growth of contaminants (Figure 1).

### Statistical Analysis

Demographic characteristics (age, sex, ethnicity, education, marriage, BMI), health/behavioral factors (HIV, diabetes, smoking, alcohol use), environmental exposures (farming, animal contact, dust season) and clinical variables (site, smear results) were assessed. Unadjusted odds ratios were calculated to compare characteristics between patients with *NTM*, *MTB* pulmonary infections to patients who were equally at risk but do not have evidence of pulmonary mycobacterial infection to determine the correlates of *NTM*, and *MTB* infections. We then compared patients with *NTM* infections to those with *MTB* infections to determine differences in characteristics. Statistical significance was determined with Fisher exact tests or chi-square tests, as appropriate. Stratified analysis was used to evaluate potential confounding and effect modification. To evaluate seasonal differences in the occurrence of *MTB* and *NTM* mycobacterial infections, we assessed the proportion of cases of each infection among all subjects screened in each month together with the 95% confidence interval of the proportions.

Multivariable logistic regression was then used to adjust for confounding and effect modification in comparing the different groups. Potential covariates were manually added and removed one-by-one to develop the best model. A variable was retained in the model if it was significant (p<0.05), if it changed the magnitude of coefficients for other variables by >20%, or if it was considered an important covariate due to biologically plausible relationships. Combinations of covariates were examined for potential effect modification based on results of the stratified analysis and by testing interaction terms. Statistical analysis software (SAS Institute, Inc., Cary, North Carolina) version 9.2 was used for the analysis. Two-sided *p-values* of 0.05 or less were considered statistically significant.

## Results

Of the 1,603 participants enrolled 1,391 (87%) were from the NTBLTC, Zaria, the rest 212 (13%) were recruited at BDH, in Kaduna City. The mean age in years and standard deviation (SD) of the study sample was 37.0 (13.8) and 43.8% were females. The mean body mass index (SD) was 19.2 (4.6). About 52% of the study population were underweight (BMI<18.5) according to the international WHO criteria for BMI. Participants were predominantly of the Hausa-Fulani ethnic 1,252 (78%) and the majority (980; 61%) had a formal education level of junior secondary school (8^th^ Grade) or lower. A summary of characteristics of the study population stratified by site is provided in Table 1.

**Table 1:** Measured demographics and laboratory characteristics of participants stratified by site.

Characteristics	NTBLTC at Zaria*n = 1391 (%)*	BDH at Kaduna*n = 212 (%)*
Age: Mean (SD)	37.0 (13.9)	36.6 (13.5)
BMI: Mean (SD)	19.0 (4.6)	20.8 (4.4)
Female gender	591 (42.5)	111 (52.6)
Education: 8^th^ Grade or less	900 (64.7)	80 (37.9)
Married	991 (71.2)	122 (57.8)
Hausa or Fulani Ethnic group	1148 (82.5)	104 (49.3)
Prevalence of HIV	312 (22.4)	66 (31.3)
Prevalence of *MTB* complex infection	315 (22.6)	60 (28.3)
* Mycobacterium tuberculosis*	298 (21.4)	56 (26.4)
* Mycobacterium africanum*	16 (1.2)	4 (1.9)
* Mycobacterium bovis*	1 (0.0)	0 (0.0)
Prevalence of *Non-tuberculosis Mycobacterium (NTM)* infection	59 (4.2)	10 (4.7)
* Mycobacterium intracellulare*	16 (1.2)	5 (2.4)
* Mycobacterium abscessus*	8 (0.6)	0 (0.0)
* Mycobacterium fortuitum*	4 (0.3)	0 (0.0)
* Mycobacterium sucrofulaceum*	3 (0.2)	0 (0.0)
* Mycobacterium gordonae*	4 (0.3)	0 (0.0)
* Mycobacterium malmoense*	2 (0.1)	0 (0.0)
* Mycobacterium kansasii*	0 (0.0)	1 (0.5)
* Mycobacterium interjectum*	1 (0.0)	0 (0.0)
* Mycobacterium peregrinum*	1 (0.0)	0 (0.0)
* Mycobacterium xenopi* * Not characterized*	1 (0.0)19 (1.4)	0 (0.0)4 (1.9)

There were 444 (28%) cases diagnosed with pulmonary mycobacterial infections, 22 (1.4%) cases had pulmonary infection with other AFB agents that were not of the genus mycobacteria (NMY). Samples from 234 (14.6%) cases were contaminated and 903 (56%) samples of clinically symptomatic patients were without any detectable isolates by the study testing algorithm.

Of the 444 cases of pulmonary mycobacterial infections, 375 (84.5%) were confirmed as *MTB*. Of these, 354 cases (94.4%) were *M. tuberculosis*; 20 cases (5.3%) were *M. africanum*; and one case (0.3%) was *M. bovis.* The remaining 69 (15.5%) mycobacterial cases were determined to have *NTM* isolates. For 23 (33.3%) of these, the *NTM* species were not characterized by the GenoType Mycobacterium *CM/AS* assay while the remainder were divided between *M. intracellulare* 21 (30.4%), *M. abscessus* 8 (11.6%), *M. fortuitum* 4 (5.8%), *M. sucrofulaceum* 3 (4.3%), *M. gordonae* 4 (5.8%), *M. malmoense* 2 (2.9%), *M. kansasii* 1 (1.4%), *M. interjectum* 1 (1.4%), *M. peregrinum* 1 (1.4%) and *M. xenopi* 1 (1.4%) species. Mixed infection of *NTM* and *MTB* was found in one case (0.2%). One-hundred-one (27%) of the cases with *MTB complex* isolates, 26 (38%) of cases with *NTM* isolates, 9 (41%) of those with *NMY* isolates and 185 (20%) of cases without any detectable isolates have co-infection with HIV.

### Correlates of Mycobacterial Infections

#### Non-tuberculous mycobacterium

Compared to cases without any detectable isolates, individual with *NTM* infections were more likely to be older than 35 years (58% vs. 46%; p = 0.05), present during the Harmattan dust season (48% vs. 23%; p =  <.0001) and have co-infection with HIV (38% vs. 21%; p = 0.0009). The relationship between HIV and *NTM* differed significantly across the two strata of age (25% vs. 19%; p = 0.38 in those over 35 years and 55% vs. 22%; p =  <.0001 in those aged 35 years or less; *p* interaction = 0.03). This modification of the effect of HIV on *NTM* by age is presented in the form of two adjusted group specific multivariable logistic regressions in Table 2. After adjusting for all other variables those aged 35 years or younger were more likely to be infected with HIV compared to those older than 35 years.

**Table 2:** Adjusted multivariable logistic regression analyses of correlates of *NTM* infection by age group among patients visiting two tuberculosis treatment sites in northern Nigeria from August 2010 to July 2011.

		Age >35 years			Age ≤35 years	
	*OR*	*95% CI*	*p*	*OR*	*95% CI*	*p*
HIV infection	1.40	[0.65–3.00]	0.39	3.76	[1.72–8.22]	0.00
Harmattan season	2.74	[1.42–5.30]	0.00	1.79	[0.82–3.91]	0.14
BMI >19.2	0.59	[0.30–1.15]	0.11			
Alcohol intake				2.80	[1.14–6.87]	0.02

*NTM*: non-tuberculous mycobacterial infection.

BMI: Body Mass Index.

#### Mycobacterium tuberculosis complex

Cases with *MTB complex* infections in comparison to those without any isolates were more likely to have HIV co-infection (27% vs. 21%; p = 0.01), present during the Harmattan dust season (29% vs. 23%; p = 0.003) use alcohol (17% vs. 11.2; p = 0.003) and smoke cigarette (28% vs. 17%; p = ,<.0001). They were however, less likely to be older than 35 years (30% vs. 46%; p =  <.0001), belong to female sex (34% vs. 47%; p =  <.0001) and have BMI of 19.2 or less (33.6% vs. 49.4%; p = <.0001). After adjustment in a multivariable logistic regression that included in addition, patients’ ethnicity and study site, *MTB* infection was significantly associated with HIV co-infection (OR, 1.7, 1.2–2.5; p = 0.04), cigarette smoking (OR, 2.0, 1.5–2.7; p =  <.0001) and age below 35 years (OR, 2.0, 1.7–2.6; p =  <.0001).

#### Pattern of occurrence: NTM versus MTB complex

**Figure 2:**
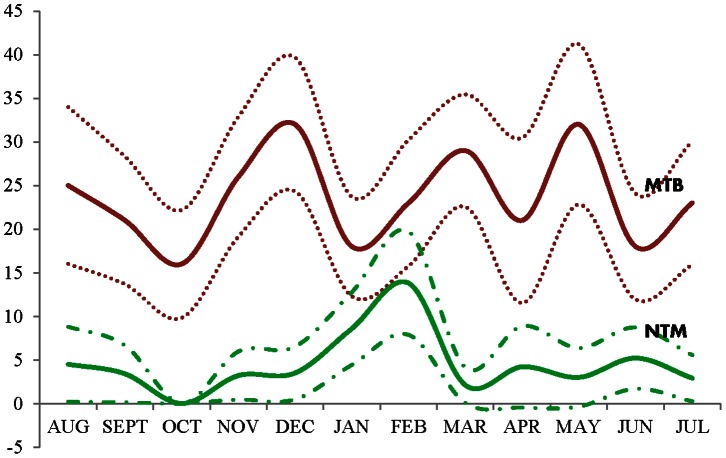
Pattern of occurrence of tuberculosis and non-tuberculous mycobacterial infections over 12 calendar months in Nigeria. Y-axis: monthly proportions of NTM and MTB complex among all subjects screened (solid lines indicate proportion while dotted lines are the 95% confidence interval of the proportion) X-axis: time in months.

At the univariate level, cases with NTM differed significantly from those with MTB as regards age, standard of care smear results, and seasonal occurrence (Table 3). The monthly proportion of patients with NTM infection was highest at the peak of the Harmattan dust season (January to February). The MTB complex infections, on the other hand, peaked at several time periods without a unique pattern of occurrence through the twelve calendar months (Figure 2). In the multivariable analysis, the adjusted odds of HIV infection (although not statistically significant) and of entry into the study during the dust season were both higher for NTM cases but the odds for having a positive standard of care smear test were less for the NTM cases compared to the MTB complex patients (Table 3).

**Table 3:** Comparison of characteristics between *NTM* and *MTB Complex* cases visiting two tuberculosis treatment sites in northern Nigeria from August 2010 to July 2011.

Characteristics	*NTM* N = 69		*MTB Complex* N = 375		OR	95% CI	*a*OR	95% CI
	n	%	n	%				
HIV infection	26	37.8	101	26.9	1.64	[0.96–2.81]τ	1.46	[0.79–2.70]
Female sex	30	43.5	126	33.6	1.52	[0.90–2.56]τ		
Age >35 years	40	58.0	113	30.1	3.20	[1.89–5.41]**	2.77	[1.52–5.02]*
BMI ≤19.2	31	44.9	126	33.6	1.61	[0.96–2.71]τ		
Farming	23	33.3	107	28.5	1.25	[0.72–2.17]		
Keep livestock	20	29.0	86	22.9	1.37	[0.77–2.43]		
Majority ethnic group	50	72.5	284	75.7	0.84	[0.47–1.50]		
Alcohol intake	12	17.4	65	17.4	1.00	[0.51–1.97]		
Cigarette smoking	15	21.7	105	28.0	0.71	[0.39–1.32]		
History of diabetes mellitus	2	3.0	17	4.6	0.64	[0.14–2.84]		
SoC smear test positive	5	7.3	229	61.1	0.05	[0.02–0.13]**	0.06	[0.02–0.14]**
Harmattan dust season	33	47.8	107	28.5	2.30	[1.36–3.87]*	2.34	[1.28–4.29]*

*NTM*: Non-tuberculous mycobacterial infection.

*MTB complex:* Mycobacterium tuberculosis complex.

OR: unadjusted Odds ratio.

*a*OR: adjusted odds ratio (multivariable logistic regression analysis).

CI: confidence interval.

τ *P*<.10;

* *P*<. 05;

** *P*<.0001.

SoC: Standard of care.

## Discussion

An unexpectedly high number of cases who sought clinical treatment for tuberculosis in Nigeria were caused by *NTM* infection (15%). A relationship between *NTM* cases and the period of the Harmattan dust was seen both in the regression analysis and the temporal analysis. Harmattan is a West African trade wind that occurs during the winter and is characterized by heavy amount of dust in the air, low humidity and reduced visibility. [18] This wind has been previously associated with outbreaks of bacterial meningitis (meningococcal meningitis) in the West African sub-region, the largest in recent times being the 1996 epidemic. [19], [20] Airborne fungi have also been isolated directly from Harmattan dust in Zaria [21] and cases of pulmonary blastomycosis with TB-like features were reported in the past. [22]. Higher risks for environmentally acquired pulmonary mycobacterial infections have been previously reported for individuals with occupational exposures to dust [12], [23], [24].

In addition to serving as a source of exposure to environmental mycobacteria, the intense air pollution and the irritant effect of heavy doses of coarse and fine particulates on the mucosa as well as alveolar macrophages could aid invasion and infection by pathogens suspended in the dust. Of all dust types, silica dust, consisting of fine crystalline quartz carries a higher risk of predisposition to infection by opportunistic *Mycobacteria*. [25], [26] Quartz (silicon oxide) binds to alveolar macrophages (a key component of pulmonary host defense system) to trigger a chain of reactions that destroy the macrophages making the host vulnerable to infection by less virulent pathogens. [27], [28].

Although the temporal pattern, comparative difference with *MTB complex* cases and biological properties of the Harmattan dust suggest a role for it in the spread of these known environmental pathogens, our study is a cross-sectional study and so it is not possible to determine causality. It would be difficult to follow a sufficiently large cohort of uninfected individuals over time to detect incidence associated with the Harmattan season, but further research is needed to better understand this risk.

*Non-tuberculous mycobacterial* infections share clinical and radiographic similarities with *MTB.* They tend to be more common among older age groups, people with pre-existing lung conditions, cases of advanced HIV disease and may take long to treat often with poor outcome compared to *MTB* [29], [30], [31]. The disease was also reported to be higher among farmers and affect different anatomical site among persons of Caucasian race or ethnicity. [32] In this study we found that *NTM* infected cases were older in age compared to *MTB*-infected cases, and the disease was commoner among younger patients with HIV. However, we did not find any association between *NTM* infection and ethnicity or farming. Given the very low sensitivity of the standard of care smear diagnostic test and absolute specificity of the newly WHO recommended point-of-care diagnostic test (GeneXpert) for the *NTM*, a mechanism for the routine identification of *NTM* infections in high burden resource limited areas of the world is urgently needed [33]. Since the current standard of care does not include bacterial characterization then some *NTM* cases with positive smears will continue to be misclassified as *MTB* and receive conventional TB therapy to which some of the *NTM* species may be resistant, and a large majority of *NTM* infections will remain undetected. In fact, *Mycobacterium intracellulare*, the most common *NTM* strain characterized in this study has no more than an estimated 50% long term favorable response to conventional TB therapy [29]. The diverse *NTM* species characterized may mean multiple treatment options or combinations. The *NTM* cases undetected by standard of care are likely to be treated for respiratory infections other than TB with increased tendency for treatment failures and drug resistance development.

The majority of *MTB complex* cases were caused by *M. tuberculosis* with a few cases caused by *M. africanum*, and only one case by *M. bovis*. The proportion of *M. africanum* tuberculosis is lower compared to a previous report from southern Nigeria that found a 13% prevalence of *M. africanum* infection among pulmonary TB *cases*. [34] Studies from other west African nations have found 9% to 28% of *MTB* isolates were *M. africanum*. These studies also reported a very low prevalence of *M. bovis* pulmonary TB in the neighboring west African countries of Ghana (3%), Mali (0.8%), Cameroun (0.2%), and Burkina Faso (0%) [35], [36], [37], [38]. The single case of *M. bovis* infection in this study was in an HIV co-infected patient. *Mycobacterium bovis* infection generally results from spread from livestock or their products and may be transmitted through routes other than the respiratory system. Sampling suspected cases of cervical or abdominal TB might have yielded a different outcome.**.**

Our study is a cross-sectional study and hence limited in its ability to adequately evaluate causal and temporal associations between HIV, dust exposures and mycobacterial infections. The findings may only represent facility-based TB patients whereas others who do not visit these healthcare facilities may differ. However, bias in this study was minimized by giving every suspected case visiting the facilities for the first time a chance to participate in the study. While we opted to culture only one out of the three sputum samples, namely the early morning sample, this is unlikely to have reduced our ability to detect positive cases, given that the early morning sample is known to have the highest concentration of mycobacteria in patients, and it also reduces the likelihood of contamination that can occur as a result of smears taken from the other two samples during the clinic’s standard of care. Furthermore, a single sample liquid broth culture is reported to have a yield comparable to that from combining three sputum samples using solid cultures. [39] This is in addition to the established superiority of the Hain molecular line probe assays performed on cultured specimens over the conventional methods for identification and characterization of Mycobacterial isolates. [40], [41], [42].

### Conclusions

In conclusion, the high prevalence (15%) of clinical pulmonary TB due to *NTM* linked to Harmattan dust exposure, and to HIV co-infection (38%) presents a novel public health challenge, which needs to be considered when planning for prevention and treatment of these patients and understanding the efficacy of the standard TB regimens since their responses to these regimens are known to vary from *M. tuberculosis*. The comparable ineffectiveness of the standard of care smear test in identifying the *NTM* (environmentally acquired) infection, underscores the need for a cheaper, easier, highly sensitive and highly specific TB screening algorithm for effective disease control. Introduction of molecular detection and screening assays that include rapid detection of *NTM* infections in high burden resource limited settings should be a priority for strengthening the public health response.
